# Not Part of the Temporal Lobe, but Still of Importance? Substantia Nigra and Subthalamic Nucleus in Epilepsy

**DOI:** 10.3389/fnsys.2020.581826

**Published:** 2020-12-02

**Authors:** Sonja Bröer

**Affiliations:** Faculty of Veterinary Medicine, Institute of Pharmacology and Toxicology, Freie Universität Berlin, Berlin, Germany

**Keywords:** epilepsy, basal ganglia, subthalamic nucleus (STN), substantia nigra (SN), seizure, propagation, focal therapy, GABA

## Abstract

The most researched brain region in epilepsy research is the temporal lobe, and more specifically, the hippocampus. However, numerous other brain regions play a pivotal role in seizure circuitry and secondary generalization of epileptic activity: The substantia nigra pars reticulata (SNr) and its direct input structure, the subthalamic nucleus (STN), are considered seizure gating nuclei. There is ample evidence that direct inhibition of the SNr is capable of suppressing various seizure types in experimental models. Similarly, inhibition via its monosynaptic glutamatergic input, the STN, can decrease seizure susceptibility as well. This review will focus on therapeutic interventions such as electrical stimulation and targeted drug delivery to SNr and STN in human patients and experimental animal models of epilepsy, highlighting the opportunities for overcoming pharmacoresistance in epilepsy by investigating these promising target structures.

## Introduction

Epilepsy has afflicted mankind since the beginning of recorded history and is still one of the most common disorders of the central nervous system. In 2015 1.2% of the US population reported to suffer from epilepsy (Zack and Kobau, 2017). The causes of epilepsy are diverse and range from (1) symptomatic epilepsy, in which an underlying disease such as a brain tumor is causative for the seizures, to (2) idiopathic epilepsy, in which genetic factors that trigger epilepsy are discussed or (3) cryptogenic epilepsy if neither a pathological nor genetic cause can be determined (Engel, 2006). Epilepsy is defined by the chronic, spontaneous recurrence of seizures. Acute seizures can be triggered by various stimuli in healthy humans and animals. A seizure is a temporary phase of abnormal, excessive or synchronous activity in the brain (Fisher et al., 2005). The severity varies depending on the localization of the seizure focus in the brain and the local or generalized spread of seizure activity. A precise classification of the seizure type, the cause, the age of epilepsy onset, triggering factors and electroencephalographic findings are essential for successful therapy. Focal seizures are distinguished from generalized seizures (Berg et al., 2010). Focal seizures occur only in defined brain regions, while in primary generalized seizures the seizure activity spreads over both brain hemispheres almost immediately. About 60% of newly diagnosed epilepsy is characterized by focal seizures that originate in the mesial temporal lobe, but these focal seizures often generalize secondarily (Banerjee and Hauser, 2008).

## Current Treatment Options and Response

Patients with epilepsy experience spontaneous recurrent seizures that are treated symptomatically with systemic antiseizure medications; however, up to two thirds of these patients fail to achieve seizure freedom and are therefore classified as having a pharmacoresistant disease (Leppik, 1992; Schmidt and Löscher, 2005; Rogawski and Holmes, 2009). Despite approval of several new antiseizure medications, this proportion has not significantly changed over the past decades (Löscher and Schmidt, 2011). Consequently, there is an urgent need for new therapeutic strategies to treat this devastating condition.

For pharmacoresistant patients the best therapeutic strategy is often surgical resection of the epileptic focus, which led to seizure freedom in a clinical trial in about 60% of patients 1 year post-surgery, compared to 8% in the pharmacotherapy only group (Wiebe et al., 2001). In addition, the surgical patients rated their quality of life significantly higher; however, it is important to note that patients continued to take their antiseizure medication after resection surgery. Resection is not suitable for every patient due to a multifocal origin of the epileptic seizures (Nilsen and Cock, 2004). Other patients decline a surgical treatment because of the inherent risks associated with invasive brain surgery.

## Targeted Strategies to Block Initiation

A viable approach to treat this pharmacoresistant population could be to develop interventions that target structures that are key regulators of seizure generalization. Silencing or stimulating these defined brain regions may be achieved by pharmacological, electrical or optogenetic manipulation, ablative approaches and/or neurotransplantation of inhibitory cells, which will be discussed in the following sections. Most efforts have focused on the hippocampus since it is the seizure onset zone for 80% of seizures in mesial temporal epilepsy (mTLE) (Tatum, 2012). Targeted therapies aim to silence the hippocampus, but it has proven exceedingly challenging to model in animals. The primary issue is that traditional systemic chemoconvulsant status epilepticus animal models do not present with clear hippocampal onset of epilepsy – or they display more widespread neuropathological changes than observed in a clinical patient population (Sloviter, 2009).

## Targeted Strategies to Block Propagation

If the focus is situated in an area that is not surgically accessible or if the patient has multiple foci, a targeted approach to the epileptic focus does not present a viable option. In these cases, targeting areas of seizure propagation might be a feasible approach. As such the basal ganglia have been known for more than 30 years to play a pivotal role in seizure gating (Gale, 1988). The basal ganglia are a group of subcortical nuclei in the fore- and midbrain, which include the substantia nigra, the striatum, the globus pallidus externus, the globus pallidus internus and the subthalamic nucleus. These regions are anatomically and functionally connected to each other and to the limbic system. Changes in this network can lead to complex neuropsychiatric symptoms, cognitive changes, changes in behavior and hypo- or hyperkinetic movement disorders, as experienced by patients suffering from Parkinson's disease or Huntington's disease. Physiologically, the basal ganglia perform a filtering role, the so-called “Gating” function by selecting for desired movement patterns and inhibiting undesired activation patterns, including seizure activity in epilepsy.

## Epilepsy and the Basal Ganglia

**Figure 1:**
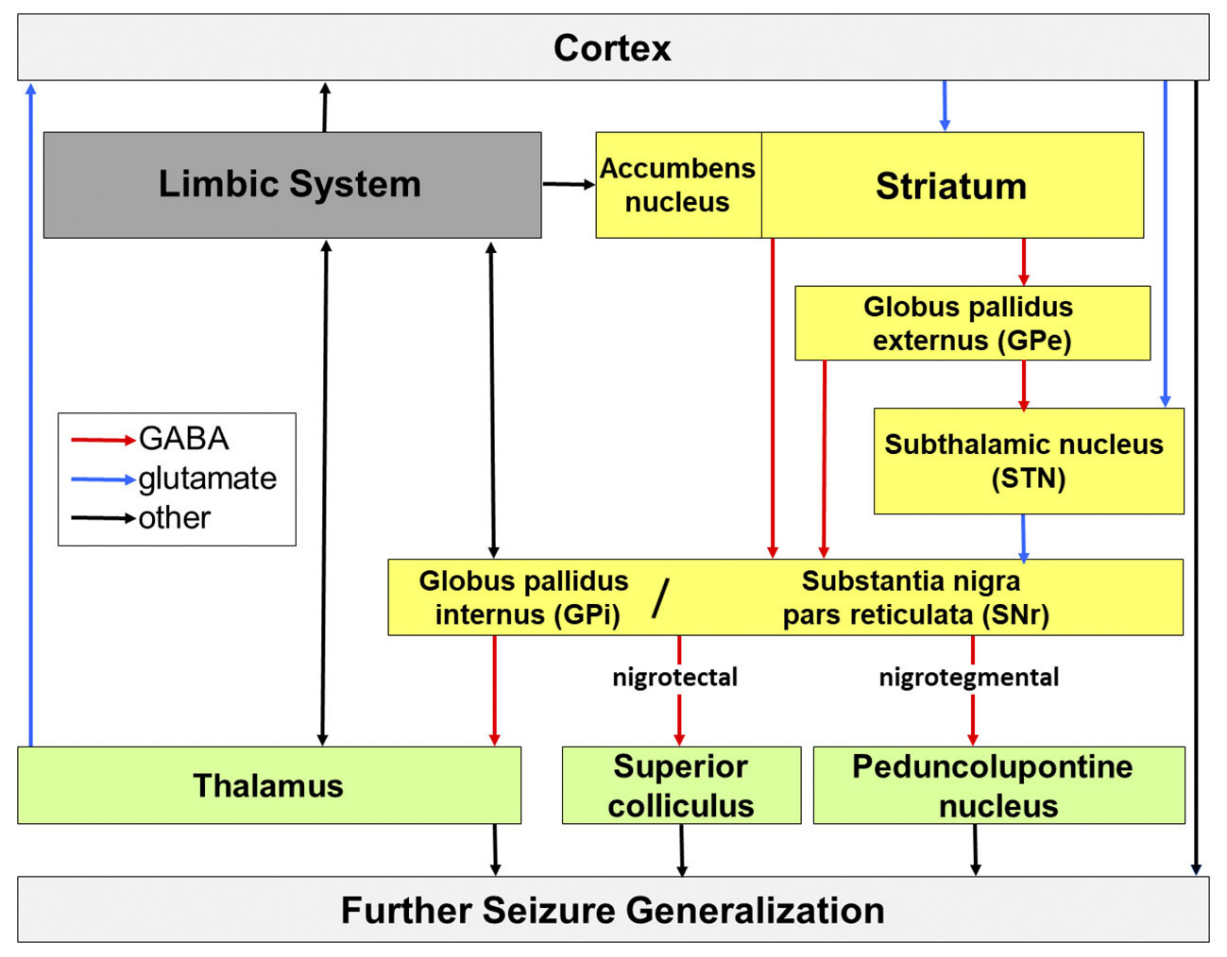
Potential routes of seizure propagation in temporal lobe epilepsy, modified from Löscher et al. (2008). Seizures that arise in the limbic system can be propagated via the cortex, basal ganglia (yellow), thalamic nuclei and midbrain and brain stem nuclei and generalize across the whole brain. Red, GABAergic transmission; blue, glutamatergic transmission; black, other chemically composite pathways.

The subcortical nuclei of the basal ganglia and their simplified interconnections are depicted in Figure 1. Regarding experimental studies in rodents, the anatomical nomenclature is slightly different than in humans. The globus pallidus in rats is known as globus pallidus externus in humans; the entopeduncular nucleus in rats is equivalent to globus pallidus internus in humans.

The interconnection of the basal ganglia consists of control loops running in parallel from the cortex to the brainstem and thalamus and from the thalamus back to the cortex. The entrance structure of the basal ganglia is the striatum, which receives input from associative, sensorimotor and limbic cortices, thalamus and the substantia nigra pars compacta (DeLong, 2000). Output structures are the substantia nigra and the entopenduncular nucleus that propagate activity via the core motor areas of the thalamus, the rostral colliculus (human: superior colliculus) and the pedunculopontine nucleus (Bolam et al., 2000).

Regions of specific interest to epilepsy are the basal ganglia output structures, the substantia nigra (SN), and its direct monosynaptic glutamatergic input structure, the subthalamic nucleus (STN). Research of the role of the SN in epilepsy has been pioneered by Karen Gale (Iadarola and Gale, 1982, cf. Velíšek, 2019). Compelling evidence for its role in relaying and spreading epileptic seizure activity is well documented (cf. Deransart and Depaulis, 2002, see next section). In addition to modulation of the SN inhibition via its monosynaptic glutamatergic input, the subthalamic nucleus (STN) can decrease seizure susceptibility (Robledo and Feger, 1990; Feger and Robledo, 1991). Consequently, the STN could also be of specific interest for targeted therapy; it is already an established neurosurgical target for deep brain stimulation in Parkinson's disease and could make translation to patients with epilepsy more feasible.

## Nigral Inhibitory System

The SN consists partly of a pars compacta (SNc) with densely packed, dopaminergic neurons, which are subject to degenerative processes in Parkinson's disease. There is some recent evidence for a role of dopaminergic SNc involvement in epilepsy (Bouilleret et al., 2008; Hu et al., 2020), however this review focuses on the inhibitory system involving the pars reticulata. The pars reticulata (SNr) has been attributed a seizure gating function (Gale, 1988; Gale et al., 2008). GABAergic neurons comprise approximately 90% of the SNr and these neurons receive input from the striatum via two routes. Inhibition of SNr neurons is achieved by direct, monosynaptic, mainly GABAergic projection neurons of the striatum (medium-sized spiny neurons; Hattori et al., 1973; Fonnum et al., 1978) and activation via an indirect, polysynaptic input via three nuclei (see Figure 1). The medium spiny neurons of the striatum project to the globus pallidus externus, and from there to the globus pallidus internus and SNr, as well as via a GABAergic projection from globus pallidus externus to the STN, followed by a glutamatergic projection from the STN to the SNr (Alexander and Crutcher, 1990; Robledo and Feger, 1990; Shen and Johnson, 2006; Deniau et al., 2007).

Inhibitory projections to different brain regions run from the SNr to brainstem and midbrain. In the event of an activation of this nigral inhibitory system, the downstream regions such as thalamus, the superior colliculus and the pedunculopontine nucleus are inhibited and increase the probability for triggering a seizure by synchronizing their cortical target regions (Redgrave and Dean, 1985). Synchronous activity is predeterminant of seizure initiation. Alternatively, inhibiting the SN disinhibits the downstream regions with the result of desynchronizing activity in the cortex and consequently increasing the seizure threshold. Therefore, direct inhibition of the SNr itself, or indirect inhibition of the SNr by inhibiting its direct source of innervation, the STN, can result in anticonvulsant effects (Robledo and Feger, 1990; Feger and Robledo, 1991).

In addition, reciprocal connections run from the SNr to the limbic system, so that a seizure focus regardless of its exact localization within the limbic system can be manipulated by targeting the SNr as well (Depaulis et al., 1994; Paz et al., 2007; Gale et al., 2008; Löscher et al., 2008).

## Experimental Evidence: Substantia Nigra Pars Reticulata

Overall, the local administration of a therapeutic agent, in the form of a substance, a transplant, or electrical stimulation, is far more specific than the systemic administration of a drug or compound (Boison, 2007). It was shown almost 40 years ago that microinjection into the midbrain of a GABA-potentiating drug, vigabatrin, acted as an anticonvulsant in a rat seizure model (Iadarola and Gale, 1982). Using the direct GABA_A_ receptor agonist muscimol, Gale (1985) was able to identify the SNr as the region mediating the antiseizure effect. During and before a generalized seizure nigral neurons are increasingly active (Engel et al., 1978; Nehlig et al., 1998; Dubé et al., 2000), while anticonvulsant drugs or GABA itself decrease firing of the GABAergic neurons (Bloms-Funke and Löscher, 1996; Windels and Kiyatkin, 2004). The SNr is also affected by plastic network changes in the epileptic brain, as shown in the widely used electrical kindling animal model for epilepsy (Gernert et al., 2004; Töllner et al., 2011).

Evidence from several experimental models of seizures and epilepsy have shown that increased inhibition of nigral neurons, which was achieved by locally administering GABAergic drugs into the SNr, was responsible for a reduction or suppression of experimentally triggered seizures and are summarized in Table 1 (Gale and Iadarola, 1980; Le Gal La Salle et al., 1983; McNamara et al., 1984; Garant and Gale, 1986; Turski et al., 1986; Depaulis et al., 1989; Xu et al., 1991; Zhang et al., 1991; Depaulis, 1992; Garant et al., 1995; Velíšková et al., 1996a; Smolders et al., 1997; Deransart et al., 2001; Nolte et al., 2006; Bröer et al., 2012; Gey et al., 2016). Genetically modified rats that display spontaneous absences and rats that experience genetically determined audiogenic seizures upon stimulation could also be treated successfully with injection of GABA_A_ agonists into the SNr (Depaulis et al., 1988, 1990; Deransart et al., 2001, see Table 1).

**Table 1:** Summary of selected references investigating SNr modulation in experimental seizure models.

**Modulation of SNr**	**Model/seizure type**	**Effect on seizures**	**References**
GABAergic drug	MES	Decreased duration of tonic hindlimb extension	Gale and Iadarola, 1980; Iadarola and Gale, 1982; Zhang et al., 1991
	PTZ low dose i.p.	Increased seizure threshold^1^; decreased spike-and-wave discharges (no effect on motor seizures with higher dose of PTZ)^2^	^1^Zhang et al., 1991; ^2^Depaulis et al., 1989; Depaulis, 1992
	PTZ i.v.	Increased seizure threshold	Iadarola and Gale, 1982; Bröer et al., 2012; Gey et al., 2016
	BIC i.v.	Decreased seizure duration or severity^3^	Iadarola and Gale, 1982; Garant and Gale, 1986; ^3^Iadarola and Gale, 1982
	Pilocarpine i.p.	Suppressed seizures, prevented brain damage	Turski et al., 1986
	Pilocarpine i.hc.	Partial protective effect	Smolders et al., 1997
	γ-butyrolactone i.p.	Reduced duration of spike-and-wave discharges	Depaulis et al., 1989; Depaulis, 1992
	THIP	Reduced duration of and increased latency to onset of spike-and-wave discharges; no effect in higher dose of THIP	Depaulis et al., 1989; Depaulis, 1992
	Flurothyl inhalation	Increased seizure threshold (vigabatrin and low dose muscimol); proconvulsant in high dose muscimol and THIP^4^; proconvulsant in pups^4^; anterior SNr anticonvulsant, posterior SNr proconvulsant^5^	Xu et al., 1991; ^4^Garant et al., 1995; ^5^Velíšková et al., 1996a
	Audiogenic	Suppressed spike-and-wave discharges; suppressed clonic audiogenic seizures	Deransart et al., 2001
	Kindling	Increased afterdischarge threshold (ADT)^6,7,8,9^; no effect on motor seizures^6^; suppressed motor seizures^7,9^	^6^Le Gal La Salle et al., 1983; ^7^McNamara et al., 1984; ^8^Nolte et al., 2006; ^9^Töllner et al., 2011
	Absence	Suppressed spike-and-wave discharges	Depaulis et al., 1988, 1990; Deransart et al., 2001
GABAergic cell	Pilocarpine i.p. in lesioned animals	Decreased seizure susceptibility, not clearly dependent on cell transplant	Fine et al., 1990
	Kainate i.p.	Decreased seizure susceptibility	Castillo et al., 2006
	PTZ i.v.	No effect	Backofen-Wehrhahn et al., 2018
	Kindling	Increased ADT and decreased seizure severity	Löscher et al., 1998
	Pilocarpine SE	Suppressed spontaneous seizures	Thompson and Suchomelova, 2004
	Kainate SE	Suppressed spontaneous seizures, decreased mortality	Castillo et al., 2008
	Absence	No effect	Castillo et al., 2010
Stimulation	Flurothyl inhalation	Anticonvulsant depending on SNr subregion and animal's age	Velísek et al., 2002
	Kainate s.c.	No effect	Usui et al., 2005
	Kindling	Pre-stimulation retarded kindling process, prolonged latency to or blocked motor seizures^10^; suppressed seizures in fully kindled for up to 4 days^11^	^10^Morimoto and Goddard, 1987; ^11^Shi et al., 2006
	Absence	Suppressed seizures, but repeated stimulation was ineffective or could worsen seizures	Feddersen et al., 2007
Optogenetic silencing	PTZ i.p.	Suppressed seizures (nigrotectal); worsened seizures (nigrotegmental)	Wicker et al., 2019
	BIC (piriform cortex)	Suppressed seizures (nigrotectal); no effect (nigrotegmental)	Wicker et al., 2019
	Audiogenic	Suppressed seizures; no effect (nigrotegmental)	Wicker et al., 2019
	Absence	Suppressed seizures (nigrotectal and -tegmental)	Wicker et al., 2019
Lesion	BIC i.v.	Reduced seizures	Garant and Gale, 1983
	MES	Reduced incidence of tonic seizures	Garant and Gale, 1983
	Kindling	Increased severity and duration^12^; suppressed seizures^13^	^12^Zhang et al., 2019; ^13^McNamara et al., 1984
	Kainate (low dose)	Increased seizure susceptibility and severity	Fan et al., 2000

*MES, maximum electroshock; PTZ, pentyleneytetrazole; BIC, bicuculline; THIP, 4,5,6,7-tetrahydroisoxazolopyridin-3-ol; i.p., intraperitoneal; i.v., intravenous; s.c., subcutaneous; i.hc., intrahippocampal; SE, status epilepticus. Effect on seizure findings have been summarized, specific findings are linked to the respective reference by the superscript numbers*.

High frequency stimulation has also been explored and showed comparable success in various seizure models. Deep brain stimulation of the SNr was capable of suppressing generalized and focal seizures in most models (Morimoto and Goddard, 1987; Velísek et al., 2002; Shi et al., 2006; Feddersen et al., 2007), however a study also reported no effect on kainate-induced seizures (Usui et al., 2005).

One of the disadvantages of focal drug delivery or stimulation for clinical translation is the necessity of re-administering or continuously delivering the treatment of choice. Development of tolerance has been reported for systemic GABAergic drug administration in experimental models and patients (Löscher and Frey, 1987; Löscher and Schmidt, 2006) and more recently, in a subset of animals that received a chronic focal drug delivery regimen into the basal ganglia (Gey et al., 2016). A strategy that could overcome this challenge is neurotransplantation of GABA-releasing cells that functionally integrate into the host tissue. Transplantations of inhibitory interneurons to restore the imbalance between excitation and inhibition in the epileptic network have mostly been located in the hippocampus. Due to massive anatomical restructuring that occurs in the chronic epileptic hippocampus, this is a natural target for regenerative therapies. Several studies have shown strong and long-lasting anticonvulsant effects after xeno- and allotransplantation of GABAergic cells into several chronic models of experimental epilepsy (Baraban et al., 2009; Waldau et al., 2010; Hunt et al., 2013; Cunningham et al., 2014; Henderson et al., 2014; Casalia et al., 2017; Bröer et al., 2019). These studies have in common that cells were targeted at the site of seizure initiation. Transplantation into the SNr or other seizure propagating structures has not been as widely investigated. The first studies were performed in the 1990s: After grafting fetal GABAergic cells into the SNr, the seizure susceptibility to pilocarpine decreased (Fine et al., 1990), but the effect could not be confirmed to be dependent on the release of GABA from the transplanted cells, as the graft survival was not assessed quantitatively and more importantly, transplantation of non-GABAergic cells showed a similar response. More recent studies have only shown transient anticonvulsant effects of neurotransplantation into the SNr: Grafting of fetal, striatal cells into the SNr of kindled rats increased the afterdischarge threshold and decreased severity in the kindling model (Löscher et al., 1998). In this study, the effect could be directly attributed to the GABAergic cells, since non-GABAergic cells or cell medium did not produce a similar effect. The use of genetically modified cells from cell lines that release higher amounts of GABA confirmed these results in different seizure models (Thompson and Suchomelova, 2004; Castillo et al., 2006, 2008; Nolte et al., 2008), but were without effect in absence seizures (Castillo et al., 2010, see Table 1). A possible explanation for a missing or transient anticonvulsant effect could be the low survival of cells in the SNr (Backofen-Wehrhahn et al., 2018). The basal ganglia do not experience such significant anatomical restructuring induced by epilepsy as that which occurs in the hippocampus. There is some evidence for higher persistence of grafted cells in areas of brain damage such as the hippocampus in epilepsy (Zaman et al., 2001; Zaman and Shetty, 2002), however many recent studies have shown promising long-term survival of inhibitory neurons in healthy brain regions up to 1 year after transplant, which seems to be independent from the host tissue (Southwell et al., 2012; Masnaghetti et al., 2019), justifying the continuation of researching neurotransplantation into basal ganglia as a treatment option for epilepsy.

Although anticonvulsant effects of lesioning the SNr in bicuculline-induced seizures and against electroshock seizures have been reported (Garant and Gale, 1983), it does not seem to be a viable option for seizure reduction since there is conflicting evidence on its effect on kindled seizures. Lesioning the SNr significantly increased severity and duration of kindled seizures in one report (Zhang et al., 2019), but suppressed seizures in another study (McNamara et al., 1984). A unilateral dopamine-induced lesion of the SNr facilitated kainate-induced seizures in rats (Fan et al., 2000).

There is also conflicting evidence on the SNr's uniformity in its seizure modulating properties. Gernert et al. (2004) reported that kindling resulted in neuronal plasticity within the SNr, that was subregion specific. Furthermore, a microinjection of vigabatrin into the posterior SNr resulted in proconvulsive effects in the Flurothyl model but had an anticonvulsant effect in the anterior SNr (Velíšková et al., 1996a), while both targets mediated an anticonvulsant effect in the pentylenetetrazole model (Bröer et al., 2012). Possible reasons for these contradicting results could be differences in expression of receptor types (Velíšková et al., 1998), sub-region-specific efferences, the animal model used, or the age and sex of the animals (Shehab et al., 1996; Gernert and Löscher, 2001). It is believed that different seizure types involve different output structures (Depaulis et al., 1994), for example tonic seizures could not be manipulated by nigral inhibition (Deransart et al., 2001). A recent study by Wicker et al. (2019) has utilized optogenetic silencing in order to clearly dissect the role of the output pathways of the SNr in four different seizure models. They found that inhibition of the SNr itself suppressed acute seizures induced by intraperitoneal pentylenetetrazole injection or by direct injection of bicucilline into the area tempestas of the piriform cortex. Furthermore, audiogenic seizures in Genetically Epilepsy Prone rats (GEPR-3) that respond to loud noise with convulsions, as well as absence seizures in the systemic gamma butyrolactone model were suppressed with this approach. Interestingly, selective inhibition of the projection from the SNr to the superior colliculus (nigrotectal) achieved the same effect, whereas selective silencing of the projection to the pedunculopontine nucleus (nigrotegmental) provided mixed results (cf. Figure 1), as it reduced absence seizures, but aggravated pentylenetetrazole-induced seizures, and had no effect on audiogenic and piriform seizures (Wicker et al., 2019). Newer research suggests a direct role of the basal ganglia in absence seizure initiation and proposed the pars compacta as a novel target for seizure modulation (Hu et al., 2020).

## Experimental Evidence: Subthalamic Nucleus

Compared to the SNr, the role of the STN in epilepsy is less studied but is a well-characterized clinical target for therapeutic neurostimulation in other neurological diseases such as Parkinson's disease or dystonia (Benabid et al., 2001; Benabid, 2007; Al-Otaibi et al., 2011). The surgical accessibility of this structure could make it a feasible candidate for targeted therapy in epilepsy as well (see The basal ganglia in clinical epilepsy). STN targeting might be associated with less side effects compared to SNr targeting since locomotor activation has been reported after muscimol injections into SNr, but not into STN (Dybdal and Gale, 2000). Muscimol injections into the STN lowered the activity of the SNr (Feger and Robledo, 1991) since the STN delivers monosynaptic, glutamatergic and thus excitatory input into the SNr. The first evidence for a role of the STN in promoting seizure activity was provided by Deransart et al. (1996) and Vercueil et al. (1998) in a model of genetic absence epilepsy in the rat, in which bilateral injections of GABA agonists, as well as high-frequency stimulations, suppressed spontaneous seizure activity. Similarly, an anticonvulsant effect of GABA agonist injections was shown in the kindling model (Deransart et al., 1998), in acute seizure models induced with systemic and focal application of proconvulsant drugs such as bicuculline (Dybdal and Gale, 2000) or pentylenetetrazole (Bröer et al., 2012), and after flurothyl inhalation (Velíšková et al., 1996b, see Table 2). Delivery of vigabatrin to the STN was more efficacious than delivery into the adjacent zona incerta, SNr, or striatum and its effects were comparable to systemic administration, while significantly fewer adverse events were observed with local delivery than after systemic treatment (Bröer et al., 2012). In conclusion, the inhibition of the direct striatonigral and indirect pathways play a pivotal role in the spread of epileptic seizure activity (Depaulis et al., 1994; Gale et al., 2008).

**Table 2:** Summary of selected references investigating STN modulation in experimental seizure models.

**Modulation of STN**	**Model/seizure type**	**Effect on seizures**	**References**
GABAergic drug	BIC i.v.	Protected against seizures (only bilateral muscimol, not unilateral)	Dybdal and Gale, 2000
	BIC (piriform cortex)	Protected against seizures (only bilateral muscimol, not unilateral)	Dybdal and Gale, 2000
	PTZ i.v.	Increased seizure threshold	Bröer et al., 2012; Gey et al., 2016
	Flurothyl inhalation	Reduced seizures (unilateral and bilateral muscimol)	Velíšková et al., 1996b
	Kindling	Reduced motor seizures, no effect on afterdischarges	Deransart et al., 1998
	Absence	Suppressed spike-and-wave discharges	Deransart et al., 1996
GABAergic cell	PTZ i.v.	Transient increase in seizure threshold (unilateral and bilateral)^1^; anticonvulsant effect cell-specific^2^	^1^Handreck et al., 2014; ^2^Backofen-Wehrhahn et al., 2018
Stimulation	Kainate s.c.	Reduced duration of generalized; duration of focal seizures was prolonged, but severity was decreased	Usui et al., 2005
	Absence	Suppressed spike-and-wave discharges	Vercueil et al., 1998
Lesion	Absence	Only partially effective, reduced discharge duration in 60% of animals	Vercueil et al., 1998

*PTZ, pentyleneytetrazole; BIC, bicuculline; i.p., intraperitoneal; i.v., intravenous; s.c., subcutaneous. Effect on seizure findings have been summarized, specific findings are linked to the respective reference by the superscript numbers*.

Follow-up studies assessed feasibility and efficiency of continuous microinfusion of vigabatrin into the STN and found that bilateral infusion of vigabatrin over several weeks increased GABA in the STN, leading to a significant increase in pentylenetetrazole seizure threshold. However, some animals developed tolerance to vigabatrin's anti-seizure effect (Gey et al., 2016). Furthermore, it has been described that direct stimulation of the STN is able to suppress seizures in a variety of experimental models, such as absence seizures in genetically epileptic rats (Vercueil et al., 1998), and seizures induced with systemic kainate injections (Usui et al., 2005). Two studies have focused on transplantation of GABAergic cells in the STN in an acute seizure model (Handreck et al., 2014; Backofen-Wehrhahn et al., 2018) that show transient anticonvulsant effects can be observed for a few weeks. Remarkably though, even a unilateral graft into the STN significantly affected the seizure threshold (Handreck et al., 2014). Contrary to earlier discussed options lesioning of the entire STN was not as efficient as electrical stimulation in suppressing seizures (Vercueil et al., 1998) and could be associated with increased risks, as it was reported that upon unilateral lesioning of the STN excitatory glutamate receptors were upregulated in SNr ipsilateral to the lesion, which could result in an increased susceptibility to seizures (Price et al., 1993).

## The Basal Ganglia in Clinical Epilepsy

Clinical data on the involvement and targeting of the SNr and STN in patients with epilepsy is still sparse (Vercueil and Hirsch, 2002). In 1949, Hayne et al.. reported that subcortical brain areas such as the basal ganglia can display epileptic activity simultaneously with cortical areas, but that they can also produce isolated abnormal electric activity that is independent of the cortex. A more recent study has also confirmed that generalized seizures with cortical origin lead to changed basal ganglia activity, in this case slowing of frequencies (Rektor et al., 2002). Imaging data reveal that patients with epilepsy can show atrophy of the SN and differences in blood flow, metabolism, functional connectivity and neurotransmission in the basal ganglia (cf. Semah, 2002; Keihaninejad et al., 2012; Rektor et al., 2013; Výtvarová et al., 2017).

Deep brain stimulation (DBS) of suitable brain regions, such as the STN, has been used successfully for years in the therapy of pain and movement disorders (Nguyen et al., 2011; Fasano et al., 2012). In Parkinson's Disease stimulation of the STN or the Globus pallidus internus are known to reduce motor symptoms such as tremor, bradykinesia, rigidity, and dyskinesia, but the mechanism of action of DBS on movement disorders remains under investigation (Lozano and Eltahawy, 2004). It was also the first targeted treatment that was evaluated in patients with epilepsy. Stimulations in patients have been performed in several studies in the beginning of the 2000s: The first patient was a 5-year old girl with drug-resistant and inoperable epilepsy, who received unilateral, chronic, high frequency stimulation into the STN. Her seizure frequency and severity decreased by 80% at the 2.5-year follow-up. Improvements in motor and cognitive performance were also reported (Benabid et al., 2002). Other studies have examined STN stimulation in small groups of patients and confirmed the finding that seizures were significantly reduced by 50% in most cases (Loddenkemper et al., 2001; Chabardes et al., 2002; Handforth et al., 2006; Lee et al., 2006). Vesper et al. (2007) stimulated in the STN and SNr simultaneously and found a seizure reduction in a patient with myoclonic epilepsy, who was non-responsive to prior vagus nerve stimulation and drug treatment. In a similar case report by di Giacopo et al. (2019) stimulation of the SNr was more effective in reducing seizures than STN stimulation alone or combinatorial stimulation of both structures in a patient with myoclonic epilepsy. However, compared to the STN, there is very little clinical evidence and experience with targeting the SNr in patients. Anatomically, the SNr seems to be a less promising target as its borders are not as clearly defined as the STN's. Additionally, the SNr's larger size might make stimulation of the full structure more challenging and could result either in only a small part of the SNr being stimulated or in stimulating surrounding areas, which might lead to an increase in unwanted effects (Loddenkemper et al., 2001). In general, stimulation-associated adverse events have been reported in studies on patients with Parkinson's disease and mainly include transient hemiballism and dyskinesias, which can be controlled by adjusting stimulation parameters. Surgery-related adverse events include infections, wound dehiscence and intracranial hematomas (cf. Loddenkemper et al., 2001), but the side effects of this minimally invasive surgery may outweigh the risk of mortality from uncontrollable seizures.

Larger, double-blind and randomized clinical trials are necessary to draw conclusions regarding efficacy and safety of a treatment. One trial that started in France in 2005, the STIMEP trial, in which patients with a severe form of genetic epilepsy were recruited for STN stimulation, was terminated before full enrollment, but results have not been published at the time of this writing^1^. More clinical data is available for DBS of the anterior thalamic nucleus, which has been shown to significantly reduce seizures in pharmacoresistant patients in the SANTE trial (Fisher et al., 2010), however, the reason for seizure reduction in the temporal lobe when targeting stimulation remotely is unknown.

There are no data on drug infusion into the STN or SNr in patients with epilepsy; however a clinical trial was conducted with muscimol, a GABA agonist, infused into the seizure focus (cortex or hippocampus) of three patients (Heiss et al., 2019a). Acutely, muscimol alleviated seizures in one patient, but at the 2-year follow-up, two out of three patients were seizure-free, and one had less frequent seizures. These results can't be clearly attributed to prior muscimol infusion and thus are inconclusive. Furthermore, muscimol was not labeled and it could not be confirmed that it only distributed within the target structure. (Heiss et al., 2019a). No infusion-related adverse effects were reported. In a subsequent study, the group labeled muscimol with gadolinium, an MRI tracer, and infused it into the STN into non-human primate rhesus monkeys (Heiss et al., 2019b). Muscimol infusion did not lead to neurotoxicity, however there was a transient, dose-dependent hyperkinesia and somnolence in high doses. An earlier study had reported dyskinesias and torticollis upon infusion of muscimol into the posterior SNr, but not after infusion into the STN in macaques (Dybdal et al., 2013).

## Conclusion

The STN and SNr play a pivotal role in seizure propagation as evidenced in various experimental models of epilepsy. Early studies focused on pharmacological inhibition with GABAergic substances and provided insights into subregion-specific and model-specific effects on seizures.

It has been described that abnormal electrophysiological activity and structural changes like atrophy, blood perfusion and metabolism as well as functional changes in connectivity can be recorded from basal ganglia in patients with epilepsy. Despite these findings in patients and good preclinical evidence for successfully modulating seizures by drug infusions into basal ganglia targets, and specifically their output structures SNr and STN, very little clinical data on intracerebral drug infusion in epilepsy is available. Although GABAergic drugs have long been used for epilepsy, most are no longer protected by patents and are not seeing current development. Other challenges include the drug delivery device, the drug kinetics including distribution only within the target area and potential development of tolerance to the infused substance. On the contrary, stimulation of the STN has gained increased interest in the treatment of patients with epilepsy since it has been widely and successfully used in movement disorders such as Parkinson's disease. Available data stem from case reports and small groups of patients at single clinical sites and a large randomized clinical trial in France was terminated before complete enrollment. Cell transplantation with GABAergic cells as a restorative approach could potentially overcome some of the issues of drug delivery or stimulation if the cell product is able to persist and functionally integrate into the target structure. Further investigation is necessary to evaluate the safety, efficacy and persistence of cell transplantation into seizure propagation structures such as the STN and SNr in patients with uncontrollable seizures. All in all, modulating the basal ganglia could offer a treatment that allows for more precise targeting of seizure propagation pathways and potentially less systemic side effects for patients with epilepsy.

## Author Contributions

The author confirms being the sole contributor of this work and has approved it for publication.

## Conflict of Interest

SB is a shareholder of Neurona Therapeutics Inc.

## Footnotes

^1^https://clinicaltrials.gov/ct2/show/results/NCT00228371?term=stimep&draw=2&rank=1
